# Effects of Malaria and HIV Infection on Anemia and T-cells Levels in Children in Douala City, Cameroon

**DOI:** 10.7759/cureus.32074

**Published:** 2022-11-30

**Authors:** Charlie Ngo Bayoï, Léopold G Lehman, Calvin Tonga, Lafortune Kangam, Godlove B Wepnje, Flore C Tchanga, Minette E Tomedi

**Affiliations:** 1 Animal Biology, University of Douala, Douala, CMR; 2 Animal Biology, University of Yaoundé I, Yaoundé, CMR; 3 Zoology and Animal Physiology, University of Buea, Buea, CMR; 4 Animal Biology, University of Dschang, Dschang, CMR; 5 Aquaculture, Institute of Fisheries and Aquatic Sciences, University of Douala, Douala, CMR

**Keywords:** arv treatment, anemia, t-cells, hiv, malaria

## Abstract

Introduction

Human immunodeficiency virus (HIV) infection and malaria are priority health issues for sub-Saharan Africa. Both diseases worsen each other through their effect on the immune and hematological systems. This study aimed to determine the effects of HIV infection and asymptomatic malaria on anemia and T-cells counts in children in the city of Douala in the republic of Cameroon.

Method

From May to November 2016, 197 HIV infected and 98 HIV-free non-febrile children up to 19 years old (128 male and 167 female) participated in the study. All HIV-infected children were receiving antiretroviral treatment and co-trimoxazole. Malaria diagnosis was performed using Giemsa-stained thick blood film; immunological and hematological parameters were assessed through a flow cytometer and an automated analyzer respectively. Chi-squared or Fischer's exact tests was used to compare the proportions, Mann-Whitney and ANOVA tests were used for the means. Statistical significance was set at p˂0.05.

Results

The prevalence of malaria was 8.8%, and that of anemia was 40.7%. CD4^+^-T cells were higher in malaria-infected children, both in HIV positive and negative (p=0.049). No significant association was found between malaria parasitemia and CD8^+^-T cell levels, both in HIV-positive and negative children (p=0.41). Anemia was higher in HIV-positive children (p=0.019), especially in those with severe immunosuppression (p=0.001) and in younger children (p=0.0083). Children on HIV treatment presented lower malaria prevalence (8.6% versus 10.10%), though the difference was not significant (p=0.7068). Malaria infection was associated with lower hemoglobin levels (10.5±1.7 versus 11.2±1.4; p=0.016).

Conclusion

Malaria infection may enhance CD4^+^-T cells. Both malaria and HIV infection lead to a drop in hemoglobin levels. The HIV treatment protocol may reduce malaria prevalence.

## Introduction

Malaria and human immunodeficiency virus (HIV) infection are serious health problems mostly affecting Sub-Saharan Africa, where both diseases overlap and claim high tolls [1, 2]. In 2017, an estimated 219 million malaria cases and 435,000 deaths were recorded worldwide. Children under five represent the most vulnerable group; they accounted for 61% of all malaria-related death worldwide in 2017 [1]. On the other hand, out of the 38 million people living with HIV (PLHIV) worldwide, children up to 19 years old were estimated at 2.78 million, and about 120,000 of them died of AIDS-related causes [2]. 

There is evidence that malaria and HIV infection worsen each other [3]. In non-pregnant adults, HIV increases the risk of uncomplicated malaria, severe malaria, as well as treatment failure. HIV is also related to a higher malaria transmission rate. In pregnant women, HIV enhances malaria parasitemia and congenital transmission of the malaria parasites. In children, HIV heightens malaria morbidity and parasitemia. In return, malaria is responsible for increased HIV replication [3]. Interactions between these diseases may be due to their effects on the immune and hematological systems. HIV infection is responsible for a gradual depletion of CD4+-T cells and expansion of CD8+-T cells [4], which play a key role in the immune response against the blood stages of malaria parasites [5]. Studies concerning malaria's impact on the level of CD4+-T cells are still controversial [6, 7]. This level may depend on the activation of different subsets of T helper cells in response to malaria infection. Higher parasitemia may lead to high levels of regulatory T cells (Tregs) [8]. Elsewhere, asymptomatic malaria-infected children have presented higher levels of Th1 and Th17 cells [9].

Anemia is a direct cause of mortality, especially in severe cases [10]. During the schizogonic phase, malaria parasites invade and disrupt red blood cells, therefore causing anemia [11]. This hematological disorder is also reported in the progression of HIV infection. HIV infects stroma cells and causes a deficit in cytokine production, which has effects on the hematopoiesis process through its influence on erythropoietin concentration. Acquisition of neoplasm to bone marrow caused by HIV itself, myelodepressive chemotherapies like zidovudine, or production of autoantibodies due to chemotherapy, are the common factors leading to the reduction of red blood cells [12].

Asymptomatic malaria parasite infection is usually unnoticed, as laboratory examination is often prescribed for persons presenting with clinical symptoms of the disease. More than just reservoirs of parasites contributing to malaria transmission [13], asymptomatic carriers can also suffer the consequences of parasite development in their bodies, and co-infection with HIV may worsen their condition. Understanding malaria and HIV co-infection will be of great interest for better monitoring in places where both diseases are endemic. 

In Cameroon, malaria remains a health issue in all of the ten regions of the country; it is responsible for up to 6000 deaths per year, *Plasmodium falciparum* being the cause of 95% of cases [14]. On the other hand, about 500,000 persons aged 15 to 64 years were infected with HIV in 2018; the prevalence is 5.0% in women and 2.3% in men, with the highest prevalence in women aged 40-44 and men aged 50-54. HIV prevalence was estimated at 0.2% in children up to 14 years old and 0.7% in those aged 15-19 years old [15]. Some studies on malaria and HIV co-infection have been conducted in Cameroon [16, 17], but few investigated malaria and HIV in children in relation to immunological and hematological parameters [18]. This study aimed to assess asymptomatic malaria and HIV infections and their effects on anemia and T-cell levels in children.

## Materials and methods

Study sites

This cross-sectional study was conducted from May to November 2016 in Douala, the economic capital city of Cameroon. Patients were recruited from the Laquintinie Hospital and the Society for Women and AIDS in Africa (SWAA), two HIV treatment centers in the city.

Study design and population

Upon parental consent and their personal assent, HIV-infected children up to 19 years old with no other known chronic disease, and their negative mates (brothers, sisters, or relatives living in the same environment), were enrolled in the study.

A questionnaire was used to collect information on personal and environmental characteristics (age, sex, environment, and habitat), possession and use of mosquito nets, and other malaria prevention methods. Participants were grouped into HIV-positive and HIV-negative.

Ethical considerations

Ethical clearance was obtained from the Institutional Review Board of the University of Douala. An authorization was issued by the Regional Delegation of Public Health. Written informed consent form was obtained from parents/guardians for each of the children, and every parent was free to withdraw their child at any moment. Also, verbal assent was obtained from children above five years.

No financial retribution was proposed; however, transportation fees were supported by the research team for participants living far from the sample collection site. Information collected from participants was handled only by members of the research team and health staff in accordance with confidentiality principles. Laboratory results were shared with health staff for appropriate case management.

Laboratory procedures

Prior to sample collection, armpit temperature was measured for each child. Blood samples were collected in 5ml Ethylenediaminetetraacetic acid (EDTA), containing Vacuette® blood collection tubes (Greiner Bio-One, Kremsmünster, Austria). The blood was used for HIV a test, hematological analysis, cell count, and malaria diagnosis.

HIV Test

Each child of unknown HIV status was screened for HIV infection using the Alere Determine™ HIV 1/2 Ag/Ab Combo immunochromatographic test (Abbott Laboratories, Chicago, Illinois), a test for simultaneous and separate qualitative detection of free HIV-1 p24 antigen and antibodies to HIV-1 and HIV-2. Practically, 10µL of whole blood was applied to the sample pad, and the result was read after two minutes. The non-appearance of a pink/red line was evidence of a negative test. No confirmation test was necessary as all the screening tests were negative.

Malaria Diagnosis

Thin and thick blood films were prepared and air-dried at room temperature; then, thin films were fixed with methanol. The slides were stained with 10% Giemsa solution for 10 minutes and examined under the oil immersion (x100) of CyScope® microscopy (Sysmex Partec, Görlizt, Germany). Malaria parasites were counted against 500 leucocytes, and parasitemia was expressed as the number of parasites per microliter (µL) of blood. A slide showing no parasite in 100 high-power fields was declared negative.

Hematology

Each blood sample collected in EDTA tubes (5mL) was homogenized and connected to the sample probe of the Sysmex XP300™ automated hematology analyzer (Sysmex, Kobe, Japan), which aspires 50µL of total blood for full hematological analysis.

Immunology

Absolute CD4+ and CD8+ count was measured using a flow cytometer (CyFlow Counter; Sysmex Partec, Görlizt, Germany). Briefly, in a test tube, 20 µL of phycoerythrine-conjugated monoclonal antibody to human CD4 or CD8 (mAb PE MEM241; Sysmex Partec, Görlizt, Germany) were mixed with 20 µL of whole blood. After homogenization and incubation for 15 minutes at room temperature, 800 µL of no-lyse buffer (Sysmex Partec, Görlizt, Germany) was added to the mixture. The content was then mixed gently, and the tube was plugged into the CyFlow® Counter for automated counting.

Statistical analysis

All the data (age, gender, long-lasting insecticide-treated net (LLIN) usage, anti-retroviral treatment (ART), and co-trimoxazole observance) were introduced into Microsoft Excel 2016 worksheet (Microsoft, Redmond, Washington). Children were classified according to their HIV status (positive and negative) and age (<6 years, 6-12 years, ≥12 years). Participants with hemoglobin (Hb) concentration lower than 11 g/dL were considered anemic, as described by Mwandianvita and al. [19]. Absolute CD4+-T cell counts were classified according to the CDC classification. For children older than six years, absolute CD4+-T cell counts were classified as high (≥500/μL, no evidence of immunosuppression), moderate (200-499/μL, evidence of a moderate suppression), and low (<200/μL, severe suppression). For younger ones (six years and below), absolute CD4+-T cell counts were classified as high (≥1000/µL, asymptomatic stage), moderate (500-999/µL), and low (˂500 µL) [20]. Frequencies were compared using Chi-squared or Fischer's exact tests, while means were compared using ANOVA (H) or Mann-Whitney (U) tests. Statistical significance was set at p<0.05.

## Results

Study population characteristics

Of the 295 children (128 males and 167 females) who participated in the study, 196 (66.4%) were HIV-positive, and 99 (33.6%) were HIV-negative. All HIV-positive children were receiving antiretroviral treatment, and 92.7% reported full observance under the supervision of their parents. Four participants did not give information (missing values) about the usage of long-lasting insecticide-treated nets. Overall, 195 (67.0%) of participants reported of always sleeping under LLIN (Table 1).

**Table 1:** Characteristics of the study population LLIN: long-lasting insecticide-treated net *Includes those who sleep at times or never sleep under an LLIN

Characteristics		Frequency	95%CI
Age group	<6 years	89 (30.2%)	25.0%-37.8%
	6-12 years	131 (44.4%)	38.7%-50.3%
	>12 years	75 (25.4%)	20.6%-30.8%
Gender	Male	128(43.4%)	37.7%-49.3%
	Female	167 (56.6%)	50.7%-62.3%
Sleep under LLIN	Not always*	96 (33.0%)	27.6%-38.7%
	Always	195 (67.0%)	61.3%-72.4%
Malaria	Negative	269 (91.2%)	87.4%-94.2%
	Positive	26 (8.8%)	5.8%-12.6%
Anemia	No	175 (59.3%)	53.5%-65.0%
	Yes	120 (40.7%)	35.0%-46.5%
Human immunodeficiency virus status	Negative	98 (33.2%)	27.9%-38.9%
	Positive	197 (66.7%)	61.1%-72.3%
Immunodeficiency	Severe	19 (6.4%)	3.9%-9.9%
	Moderate	76 (25.8%)	20.9%-31.2%
	No evidence	200 (67.8%)	62.1%-73.1%

Malaria and associated factors

Malaria parasite was found in 26 (8.8%) participants (Table 1). *Plasmodium falciparum* was the only species present. None of the explanatory factors showed a significant association with malaria parasite infection in the general study population (Table 2). 

**Table 2:** Malaria parasite infection in relation to the characteristics of study participants LLIN: long-lasting insecticide-treated net *Includes those who sleep at times or never sleep under an LLIN

Characteristic		Malaria		p-value
		Negative	Positive	
Age group	<6 years	80 (89.9%)	9 (10.1%)	0.8707
	6-12 years	120 (91.6%)	11 (8.4%)	
	>12 years	69 (92.0%)	6 (8.0%)	
Gender	male	116 (90.6%)	12 (9.4%)	0.928
	Female	153 (91.6%)	14(8.4%)	
Sleep under LLIN (n=291)	Not always*	90 (93.8%)	6 (6.3%)	0.3639
	Always	175 (89.7%)	20 (10.3%)	
HIV status	Negative	88 (89.8%)	10 (10.2%)	0.7068
	Positive	181 (91.9%)	16 (8.1%)	
Immunodeficiency	Severe	19 (100%)	0 (0%)	0.0531
	Moderate	73 (96.1%)	3 (4.0%)	
	No evidence	177 (88.5%)	23 (11.5%)	

Malaria prevalence was slightly higher in HIV-free children compared to HIV-positive though the difference was not significant (10.2 versus 8.1, p=0.707; Table 3).

**Table 3:** Malaria parasite density disaggregated per HIV status and age group in relation with LLIN usage U: Mann-Whitney test; GMPD: geometric mean parasite density; SD: standard deviation; LLIN: long-lasting insecticide-treated nets *statistically significant

Characteristic			GMPD ± SD (range)		ANOVA-test	p-value
			Irregular use of LLIN	Always use LLIN		
HIV status	Negative		305±2 (112-1392)	49±2 (30-80)	9.268	0.0160*
	Positive		414±2 (128-848)	123±3 (40-398)	8.023	0.0092*
Age group	<6 years		221±2 (112-400)	178±3 (80-398)	1.159	0.695
	6-12 years		464±2 (160-1392)	67±2 (40-112)	4.521^u^	0.0335*
	>12 years		520±1 (409-649)	62±3 (30-128)	20.681	0.00013*
Overall			366±2 (112-1392)	90±3 (30-398)	15.577	0.0006*

Although the use of LLIN did not show a significant association with the prevalence of malaria parasite infection, it was significantly associated with malaria parasite densities (Table 3). Participants who reported regular use of LLIN had significantly lower geometric mean parasite density (GMPD) (p=0.0006). This observation did not vary with HIV status; however, when desegregated in age groups, it remains valid in participants aged six years and above (p=0.0335 in children aged six to 12 years and p=0.00013 in children above the age of 12 years).

As presented in table 4, malaria parasitemia was associated with higher CD4+ T-cell counts (mean cells count: 1039 versus 844; p=0.0485), both in HIV-positive and negative children. HIV infection was associated with lower CD4+ T-cells counts (mean cell count: 740±422 versus 1107±505; p=0.00001), both in malaria-infected and non-infected children (p=0.03 and p=0.000001).

**Table 4:** CD4 and CD8 T-cells counts in relationship with HIV status and malaria parasite infection #ANOVA or Mann-Whitney test; U: Mann-Whitney test *statistically significant

T-cells	HIV status	Mean cell concentration ± SD (Range)		Overall	Statistics#	p-value
		Negative malaria	Positive malaria			
CD4+ T-cell	Negative	1085±493 (237-2790)	1303±586 (583-2364)	1107±505	44.8214^U^	0.00001*
	Positive	726±429 (26-2840)	874±280 (580-1606)	740±422		
	Overall	844±480 (26-1024)	1039±465 (580-2364)		3.927	0,0485*
	Statistics#	37.52	4.6710^U^			
	P-value	0.000001*	0.0307*			
CD8+ T-cell	Negative	618±326 (182-2079)	715±332 (362-1535)	628±326	23.7^U^	0.00001*
	Positive	851±544 (113-4759)	822±364 (332-1702)	844±529		
	Overall	775±496 (113-341)	781±350 (332-1702)		0.6879^U^	0.4069
	Statistics#	23.581^U^	0.562			
	P-value	0.00001	0.454			

No significant association of CD8+ T-cells counts was found with malaria infection (p=0.4069), whereas HIV infection was associated with higher CD8+T- cells counts (mean cells count: 844±529 versus 628±326; p=0.00001). This was further confirmed in the malaria-negative group (mean cell count: 851±544 versus 618±326; p=0.00001) but not in the malaria-positive group (mean cells count: 822±364 versus 715±332; p=0.4540).

Anemia in relation to malaria and HIV infection

The prevalence of anemia was significantly higher in HIV-positive children (45.7% versus 30.6%; p=0.0185) and was increasing with immune system depletion (p=0.001). Anemia was also predominant in younger children *(p=0.0083)*. No association was found with other characteristics, such as gender or LLIN usage (Table 5).

**Table 5:** Anemia in relation to the characteristics of study participants LLIN: long-lasting insecticide-treated net *Includes those who sleep at times or never sleep under an LLIN

Characteristic		Anemia		Chi-squared	p-value
		No	Yes		
Age group	<6 years	42 (47.2%)	47 (52.8%)	9.593	0.0083*
	[6-12] years	80 (61.1%)	51 (38.9%)		
	>12 years	53 (10.7%)	22 (29.3%)		
Gender	Male	34(26.6%)	94 (73.4%)	0.142	0.706
	Female	40 (24.0%)	127 (76.0%)		
Sleep under LLIN	Not always*	59 (57.4%)	37 (38.5%)	0.280	0.597
	Always	112 (57.4%)	83 (45.6%)		
HIV status	Negative	68 (69.4%)	30 (30.6%)	5.553	0.0185*
	Positive	107 (54.3%)	90 (45.7%)		
Immunodeficiency	Severe	7 (36.8%)	12 (63.2%)	13.795	0.001*
	Moderate	35 (46.1%)	41 (54.0%)		
	No evidence	133 (66.5%)	67 (33.5%) (71.56%)		

Disaggregation in age groups revealed a significantly higher prevalence of anemia in HIV-infected children, especially in children aged six years and above (Table 6).

**Table 6:** Anemia per age groups in relation to HIV status *statistically significant

Age group	HIV status	Anemia		Chi-squared	p-value
		No	Yes		
<6 years (n=58)	Negative	22 (68.8%)	10 (31.2%)	0.667	0.4142
	Positive	58 (58.6%)	41 (41.4%)		
6-12 years (n=95)	Negative	27 (58.7%)	19 (41.3%)	4.146	0.0417*
	Positive	15 (34.9%)	28 (65.1%)		
>12 years (n=139)	Negative	19 (95.0%)	1 (5.0%)	6.272	0.0123*
	Positive	34 (61.8%)	21 (38.2%)		

Also, hemoglobin concentration was significantly lower in malaria-positive children (10.5±1.7 versus 11.2±1.4), mostly in younger ones (Table 7).

**Table 7:** Hemoglobin concentration per HIV status and age groups in relation to malaria parasite infection *statistically significant

Characteristic		Hb concentration ±SD (range)		ANOVA-test	p-value
		Negative malaria	Positive malaria		
HIV status	Negative	11.6±1.4 (6.1-17.8)	10.1±2.0 (7.7-12.7)	9.995	0.0021*
	Positive	11.0±1.3 (7.8-14.9)	10.8±1.5 (7.8-13.1)	0.449	0.5034
Age group	<6 years	10.8±1.3 (6.1-12.9)	9.4±1.7 (7.7-12.6)	9.026	0.0030*
	6-12 years	11.2±1.3 (8.0-17.8)	11.1±1.2 (8.9-12.7)	0.832	0.0454*
	>12 years	11.7±1.5 (8.2-14.9)	11.0±1.8 (7.8-13.1)	1.041	0.3085
Overall		11.2±1.4 (6.1-17.8)	10.5±1.7 (7.7-13.1)	5.909	0.0157*

## Discussion

This study assessed malaria, HIV infection, and their effects on anemia and T-cell levels in children. 

Malaria parasitemia was associated with higher CD4^+^T-cell counts. In a previous study, contrary to our finding, Lisse and al. 1994 [21] found no difference in CD4^+^ and CD8^+^ T-cell counts between asymptomatic malaria-infected persons and healthy controls. However, a recent study by De Jong and al. 2017 [9] related asymptomatic malaria to an increase in Th1 and Th 17 cells, CD4+ T cell subsets. In addition, Kirinyet, 2019 [7] found an association between low malaria parasitemia with high CD4 T-cell counts. Malaria infection may have various implications on CD4^+ ^T-cells depending on parasite load; however, there is a need for more evidence on a larger population sample.

Malaria prevalence was 8.8%, lower than other studies (64.0%, 45.74%) in Cameroon, including investigations in HIV^+^ children exclusively [16, 22, 23]. This might be due to higher LLIN usage in our study population (67.01%) compared with 45.74% found by Lehman and al. 2018 [23]. It is worth noting that a nationwide LLIN distribution and awareness campaign was organized in Cameroon in 2016, contributing to higher availability and use of LLINs.* Plasmodium falciparum* was the only species found. As reported by Antonio-Nkondjio and al. [17], *Plasmodium falciparum* is largely the most predominant species found in all parts of the country, accounting for 95% of malaria cases nationwide.

Older children (≥6 years) and those who reported irregular use of LLIN presented higher malaria parasitemia. This may be due to the fact that children not using LLIN frequently are more exposed to infective mosquito bites, parents or guardians are usually more caring with younger ones, and children below five usually sleep with their mothers [24].

Anemia prevalence was significantly higher in HIV-infected children in all age groups. This result was in line with previous studies. In Nigeria, the prevalence of anemia was more than twice higher in highly active antiretroviral therapy (HAART)-naïve HIV-infected children [25]. This was probably due to HIV's impact on red blood cells itself or the side effects of some ART, such as zidovudine [12]. The prevalence of anemia in HIV-infected children in our study was similar to that prevalence found in HIV-infected children receiving ART in Mutengene, South-West region of the country (47.3%) [16].

Children below six years old had significantly higher anemia prevalence. This was in accordance with findings by Kimbi and al. [22], who reported lower anemia prevalence in older children. This difference may be related to the immune response to malaria infection as children living in malaria-endemic areas gradually acquire a protective immunity against malaria [26]. This enables their immune system to better control the parasite, which results in a lower prevalence of malaria infection and better hematologic parameters. In our study, children with malaria were the ones presenting low hemoglobin levels.

HIV-negative children presented higher malaria prevalence, even though not significant, compared with their HIV-positive counterparts (10.2% versus 8.1%). This result is in line with that obtained from a similar study on adults in which HIV-negative participants presented significantly higher malaria prevalence compared with HIV-positive ones [27]. The low malaria prevalence in HIV-positive children in our study may be due to ART and co-trimoxazole. The latter has shown antimalarial activity, which is more pronounced in asymptomatic malaria [28]. This antimalarial effect may be due to its two components, sulfamethoxazole and trimethoprim, which target dihydropteroate synthase (DHPS) and dihydrofolate reductase (DHFR), respectively [29]. These enzymes are necessary for *Plasmodium falciparum* metabolism [30]. On the other side, some ART drugs have shown antimalarial activity, including protease inhibitors (PI; such as lopinavir, atazanavir, saquinavir, nelﬁnavir, ritonavir, tipranavir, and amprenavir) and nonnucleoside reverse transcriptase inhibitors (NNRTI; such as efavirenz and etravirine) [31]. Some of these molecules (lopinavir, ritonavir, atazanavir, and efavirenz) are used in line treatment of HIV in children in Cameroon. 

The present study presented some limitations. As a cross-sectional survey, some confounders, such as the socio-economic status, living environment, and level of education of the parents that could influence malaria infection status, were not accounted for. Also, the use of mosquito nets and adherence to ART were reported by parents, which might have led to some bias. Nevertheless, the results provide valuable information on *Plasmodium falciparum* and HIV infection, and their effects on hemoglobin and T-cell levels in children in Douala.

## Conclusions

The aim of this study was to investigate malaria and HIV infection and their effects on anemia and T-cells in children. Malaria has been related to a higher CD4^+ ^T-cell count suggesting enhanced levels of CD4 by low malaria parasitemia. Both infections were related to higher anemia prevalence. ART and co-trimoxazole seemed to decrease malaria prevalence. A better implementation of malaria preventive methods and proper anemia management on the prescription of ART and co-trimoxazole are necessary to improve HIV-infected children's health conditions and decrease malaria-related morbidity and mortality in children.
